# Supplementary material for: Are carbon emissions trading and green financial instruments synergistic? -Comprehensive quantitative research based on content analysis
Source: PLoS One. 2024 Mar 7;19(3):e0298601. doi: 10.1371/journal.pone.0298601 (PMC10919601; doi:10.1371/journal.pone.0298601)
Supplement: S1 Data — (ZIP) [file pone.0298601.s001.zip › Data/地级市Z_F工作报告.zip!/║╙▒▒/▒ú╢¿╩╨/└┤╫╘╣1⁄2╓┌║┼í╛┬φ┐╦╩2╛▌═°í┐_2015.docx]

各位代表：

现在，我代表保定市人民政府向大会作工作报告，请予审议，并请市政协委员和列席会议的同志提出意见。

一、2014年工作回顾

刚刚过去的一年，是我市面临复杂严峻形势、千方百计保增长的一年，也是抢抓京津冀协同发展机遇、主动发力促发展的一年。在省委、省政府和市委的坚强领导下，我们以开放、高端、创新、统筹、效率为统领，积极应对新变化，主动适应新常态，统筹抓好稳增长、促改革、调结构、惠民生各项工作，基本完成市十四届人大二次会议确定的目标任务。全市生产总值2757.8亿元、增长7%，一般公共预算收入178.5亿元、增长6.9%，规模以上工业增加值1007.2亿元、增长6.7%，固定资产投资2172亿元、增长15.6%，社会消费品零售总额1355.9亿元、增长12.4%，城乡居民人均可支配收入分别达到21751元和9573元、增长9.6%和12.2%，居民消费价格指数上涨1.5%，主要指标增速均高于全省平均水平。全市经济结构趋优，发展质量效益提升，人民生活持续改善。

(一)构建现代产业体系，项目建设支撑力增强。以低碳高端引领产业调整，围绕“5+2+5”产业布局[1]，制定路线图、时间表，集中优势资源要素，打造保定经济升级版。汽车产业持续做大，大王店产业园[2]、长城汽车新技术中心[3]、中汽零基地[4]等重点项目快速推进，汽车产能、技术储备再创新高，整车销售79万辆，其中长城汽车73万辆、销售收入增长11.2%，SUV销量连续12年夺冠。新能源产业回暖走升，光电产业连续7个月实现正增长，扭转了近3年持续下滑态势，英利牵手巴西世界杯，在国际上再次打响保定品牌。太行山区光伏产业应用示范带[5]建设取得重大进展，“把太行山变成太阳山”的做法受到国务院领导充分肯定。农业保持平稳增长，粮食总产503.8万吨，建成现代农业示范园区44个、总面积35.3万亩，省部级蔬菜标准园达到27个、全省第一，农业产业化率提高到65.2%，市级以上龙头企业达到277家。服务业增势强劲，占GDP比重提高1.1个百分点。旅游业总收入411.5亿元、增长29.1%，白石山、野三坡成为热点景区，白洋淀旅游人数、收入实现“十连增”。两家外埠银行入驻保定，银行机构达到26家；挂牌上市企业17家、达到44家，居全省前列；全市贷款余额增长19.3%，存贷比提高2.4个百分点，金融支撑力显著增强。围绕产业发展，强力推动项目园区建设，安排省市重点项目279项、总投资6755亿元，其中10亿元以上项目144项，总投资6165.8亿元。以“五个一”[6]为抓手，整合提升34个园区[7]，新建园区道路90公里，100个大型产业化项目完成投资400多亿元，省级以上园区达到28家，成为加快发展的重要平台。

(二)主动融入京津冀协同发展，对接合作成果显著。京津冀协同发展纳入国家战略给全市极大鼓舞，我们敏锐把握、超前研究，细密准备、反复沟通，重大诉求得到国家层面认可，一些重大项目得到省委、省政府关注支持，百度“保定”搜索结果超过1亿条，我市的社会关注度空前高涨。全市上下积极行动、全面对接，与北京丰台区、朝阳区、亦庄经济开发区、天津滨海高新区等合作取得实质性进展，光大国际、中铁电气化、中体产业等一批央企进驻，北京新发地高碑店农副产品物流园[8]签约商户5300家，白沟大红门服装城[9]首批1500家北京商户入驻，中国北方商品电子交易博览中心[10]开工建设，中关村·保定创新中心[11]、解放军总医院涿州基地[12]、同仁堂和天士力[13]等项目进展顺利。全市谋划推进对接京津项目147个、总投资5320亿元，与央企合作重点项目38项、总投资1071亿元。全面加强招商引资，成功举办“两岸四地中医药创新与发展论坛”[14]，保定·深圳招商周取得良好效果。全年实际利用外资6亿美元。

(三)全面加快城镇建设，城乡面貌明显改观。围绕打造畿辅重要节点城市和区域性中心城市，加快新型城镇化进程。交通建设取得突破性进展，京港澳高速改扩建、京昆高速北延涞水段和市区北三环东延、朝阳大街南延通车，京保交通实现扩容提速；白洋淀大道、乐凯大街南延等城星通道启动实施，中心城市框架进一步拉大；开通城乡客运一体化公交线路22条，群众出行更便捷。城市管理6S行动[15]扎实推进，市人大满意度测评提升27个百分点。加快城市综合体建设，万博广场、茂业中心成为城市新地标。三大片区回迁楼开工率97%，安置住户57%。把县城建设作为推进新型城镇化的重要着力点，实施总投资579亿元的“七个一”工程[16]，建成了一批风貌特色鲜明、文化内涵彰显的精品城建项目。涿州、蠡县、曲阳等9个县(市)建成了博物馆或名人纪念馆；易县、涞水、徐水等6县获批省级园林县城，安新、定兴、雄县荣获省人居环境进步奖；南水北调中线正式向市区供水，22座县(市)地表水厂正加紧建设；18个县城“水文章”已具雏形，一亩泉河、金线河治理一期工程完工。坚持城乡统筹发展，筹资11.2亿元推进农村面貌改造提升，415个重点村面貌一新，全省现场会在涞水召开，我市经验在全省推广。

(四)强势开展污染治理，生态环境逐步改善。强力推进压煤、抑尘、控车、减排、迁企、增绿等治霾措施，全民动员净天、净城、净村，投资近3亿元完成了电力、钢铁、水泥行业的深度治理，淘汰落后产能企业48家，淘汰改造燃煤锅炉895台，淘汰黄标车10.6万辆，全部关停西部沿山的砂石料厂，主城区实施扬尘管控、机动车限行、禁止露天烧烤、限放鞭炮、全市域禁烧秸秆，圆满完成APEC会议空气质量保障任务。狠抓生态修复工作，《山水林田湖生态修复规划》编制完成，启动实施了环首都生态过渡带建设等六项生态工程[17]。白洋淀纳入国家湖泊生态修复试点，连片美丽乡村建设[18]取得初步成效。加快城镇绿化和陆地生态走廊[19]、西部生态安全屏障建设，城镇绿地新增1.9万亩，完成造林73万亩，森林覆盖率增加1.5个百分点。

(五)深入推进改革创新，发展活力日益增强。国企改革不断深化，对市本级经营性国有资产实行了集中统一监管，成立国有资产经营公司，调整完善发展投资公司，初步建立了国资监管三层体系。对商贸流通企业实施分类管理，启动了23家企业破产程序，依棉集团、宝硕氯碱公司问题取得突破。北京银行顺利托管蠡县农村信用社，妥善化解遗留近20年的重大金融风险隐患。开展土地承包经营权确权登记颁证试点，土地承包经营权流转141.9万亩。文化体制、社会体制、供销合作社综合改革取得明显成效。全民创业再掀热潮，新增市场主体近7万户，达到32.6万户，总量全省第二。科技创新进入历史最好时期，院士工作站达到16家，各级企业技术中心、工程实验室、工程技术研究中心达到160家，高新技术企业达到154家，全省第二。涞源白石山中美科技园[20]被认定为国家级国际科技创新园，白洋淀科技城[21]完成概念性规划，我市成为全国首个创新驱动发展示范市[22]。

(六)倾心倾力改善民生，社会事业全面进步。千方百计破解收支矛盾，集中财力落实各项民生政策，用于民生支出347亿元，占全部财政支出的81.7%，比上年提高5.3个百分点，高于全省2.4个百分点。43项惠民实事基本完成。以创业促就业，新增城镇就业9.2万人，登记失业率控制在4.02%。养老、医疗、生育保险覆盖面进一步扩大，“单独二孩”政策启动实施，免费孕前优生健康体检覆盖率达到87.9%；所有县(市、区)推行了阳光低保，新农合参合率达98.7%，实施城乡医疗救助29万人次、7146万元。保障房开工3.4万套、竣工2.4万套，改造农村危房1.7万户，超额完成省定任务。改造市区小街巷24条，新改扩建农村公路1321公里。解决了70.4万农村人口饮水不安全问题。完成89个空白乡镇邮政所补建运营。实施三个层面的“教育提升三年行动计划”[23]，改造提升了一批城乡中小学校。县级公立医院综合改革实现全覆盖，引进凤凰医疗集团[24]等社会资本进入我市医疗领域，市急救中心迁建竣工并投入运行，市儿童医院、妇幼保健院等迁建项目加快推进。“文化润古城”活动丰富多彩，“善美保定三部曲”[25]话剧巡演反响强烈。启动了西大街业态调整，总督署、光园、清河道署、直隶审判厅保护利用取得突破。市体育场改造完成并向公众开放，成功举办全国跳伞锦标赛、中国·希腊女子篮球国际对抗赛、中美女子排球交流赛等重要赛事，省运会获得金牌、总分双第二。投入扶贫资金31亿元，22万人脱贫，提前实现“十二五”减贫目标，阜平、曲阳、涞水成为全省样板。安全生产形势稳定，食品药品整治取得积极成效。稳妥处置圣瑞、黄金佳非法集资和道达尔油品纠纷等问题，有效化解了一批影响社会稳定的群体事件。“一年四打、连打三年”[26]专项整治行动深入推进，平安城市监控系统实现重点路段、重点部位全覆盖，社会治安持续好转，社会和谐稳定局面进一步巩固。我市被评为五星级中国慈善城市、省内唯一的全国和谐社区建设示范市。

(七)加强政府自身建设，服务效能不断提升。群众路线教育实践活动深入开展。市本级行政审批精减170项。在全省率先实施行政权力“四份清单、一个标准”[27]，严格落实“两集中、两到位”[28]制度，全面推行“两个代办”[29]，为民服务体系实现全覆盖，我市经验在全省推广。严格落实中央八项规定和各项禁令，对办公用房、公务用车、吃空饷等开展专项治理，制度的笼子越扎越密，廉政建设和反腐败工作得到加强。坚持依法行政，自觉接受市人大法律监督、政协民主监督和社会监督，办理人大代表建议、政协提案474件，办结率、满意率100%。主动运用新媒体搭建与百姓沟通平台，市长电子信箱、短信平台、961890便民热线高效运转，全年受理群众来信来电、短信和网络留言10万余件(条)，交办率100%，办结率90%以上，微博保定粉丝7.7万人、网络问政1300多件。国防动员、民族宗教、外事侨务、妇女儿童、统计审计、史志档案、防震减灾、气象、残疾人等工作取得新成绩。

各位代表!回顾一年来的工作，我们积极适应经济发展新常态，奋发作为、扎实工作，在深化结构调整中保持了经济平稳增长，在抢抓历史机遇中明确了战略方向，在破解要素制约中积蓄了发展后劲，在深化改革创新中增强了动力活力，在服务保障民生中凝聚了人心力量，既顺应京津冀协同发展大势，谋划推动了一些利当前、惠长远、关系保定发展的“大事”，也秉承人民政府为人民的为政理念，认真解决了一大批涉及老百姓切身利益的“小事”。过去一年的成绩来之不易，这是省委、省政府和市委科学决策、正确领导的结果，是人大、政协有效监督、大力支持的结果，是全市人民齐心协力、勤奋努力的结果。在此，我代表市人民政府，向全市人民，向人大代表和政协委员，向中央、省驻保单位，向驻保部队和武警官兵，向社会各界人士和离退休老同志，向所有关心支持保定发展的海内外朋友，表示衷心的感谢和崇高的敬意!

各位代表!我们也清醒地看到，当前经济社会发展中长期积累的深层次矛盾和问题尚未根本改变，投资增长乏力，经济下行压力大，回升向好的基础还不稳固；产业层次相对较低，高端产业比重小，传统产业拉动能力下降，新兴产业还没有形成有效支撑；抓项目的力度还不够，大项目、好项目还不多；强县不强、弱县很弱，县域经济实力依然单薄；资源环境约束逐年加大，生态建设任务繁重，节能减排形势严峻；一些政府部门依法行政意识淡薄、办事效率还不高，一些公职人员为官不为、懒政怠政的现象仍比较突出，一些领域和地方腐败现象还比较严重，一些损害群众利益的问题还时有发生。

各位代表!还需要特别报告的是，环境污染尤其是空气质量不好，始终是我们的一块心病。尽管我们下了很大气力，采取了很多治霾措施，效果依然不理想。2014年，我市空气质量达标天数83天，比上年增加10天，重度以上污染天数减少14天，PM2.5年平均浓度降低了4.4%。虽然完成了省定目标，但在环保部监测的全国74个重点城市中，我市位列空气质量相对较差的前10位城市之首。尽管有基础设施薄弱、集中供热率低等多方面原因，但与政府工作力度不够、措施不到位有直接关系。作为市长，我深感不安和自责，负有主要责任。在此，向人大代表和政协委员，并向全市人民表示深深的歉意!

对以上问题，我们一定认真对待不回避，勇于担当不退缩，迎难而上、执着前行，以对人民高度负责的态度，采取更有力有效的措施，切实加以解决，决不辜负全市人民的厚望!

二、2015年主要工作任务

今年，注定是不同寻常的一年；保定，正处于跨越发展的关键时期。要看到，世界经济总体复苏疲软态势难有明显改观，我国经济新常态下多方面矛盾互相交织，全省经济转型升级、爬坡过坎的任务艰巨，我市经济下行压力没有明显减轻，“十二五”规划收官需要攻坚克难，我们面临的形势依然错综复杂，今后的工作依然充满挑战。也要看到，我市产业现状和发展方向，与国家打造经济新常态的政策取向和基本发力点相契合；京津冀协同发展战略规划落地，将使我市独特的区位优势转化为现实的发展优势；新型工业化、城镇化、信息化和农业现代化深入推进，改革开放红利持续释放，创新驱动战略深入实施，会增强弯道超车的驱动力，我们面临的机遇大于挑战，保定美好的发展未来真切可期。更要看到，经过教育实践活动深刻洗礼，随着法治保定建设深入推进，全市上下风更清、气更正、劲更足，特别是我们面临的历史机遇前所未有，各级各界对保定的关注程度前所未有，全市上下团结干事的大好局面前所未有。“唯有人民和时代不可辜负”，面对人民期盼和时代使命，我们一定担负起政府责任，汇聚强大正能量，抢抓机遇、乘势而上，用决心、信心和行动，推动保定绿色崛起，开创跨越发展新辉煌!

今年政府工作的总体要求是：全面贯彻党的十八大、十八届三中四中全会精神，认真落实省委、省政府和市委重大部署，以开放、高端、创新、统筹、效率为统领，以稳中求进、进中求快为总基调，以京津冀协同发展、创新驱动为主线，以提高经济发展质量和效益为中心，主动适应新常态，加快转方式调结构，狠抓改革攻坚，突出创新驱动，统筹城乡发展，改善生态环境，加强民生保障，努力实现打造环京津增长极的新突破。

经济社会发展的主要预期目标是：生产总值增长7%左右，规模以上工业增加值增长7%左右，固定资产投资增长16%，社会消费品零售总额增长11%，一般公共预算收入可比增长7.5%。单位生产总值能耗和化学需氧量、二氧化硫、氨氮、氮氧化物排放量、PM2.5浓度下降完成省定目标。城乡居民人均可支配收入分别增长8%和8.5%，居民消费价格指数涨幅控制在3%左右，城镇登记失业率控制在4.5%以内，人口自然增长率控制在7.8‰以内。

做好今年各项工作，要紧盯发展、稳定、民生三大任务，唱响绿色崛起、跨越发展主旋律。必须在适应新常态中寻求新突破，加强对复杂经济形势的科学研判，抓好经济运行监控，用足用活国家调控政策，确保全市经济在新常态下稳健运行，力求实现超常态发展。必须在京津冀协同发展中迈出新步伐，围绕京津冀协同发展，安排部署各项工作，找准产业发展、项目园区、生态环境、基础设施等转移与对接的突破点，统筹推进，重点攻坚，全面发力。必须在改革创新中增强新动力，注重用创新举措应对发展变化，释放改革红利，稀释转型压力；注重用市场办法解决发展问题，发挥市场主体作用，用市场机制汇聚新动能。必须在改善民生中谋求新作为，把有限财力聚焦到保障民生基本需求上，持续提升民生质量，让人民群众最大限度分享改革发展成果。必须在转变作风中树立新形象，强化法治思维，推进依法行政，持续转变作风，凝聚人心力量，切实做到干事创业、务实高效、清廉为民。

今年，作为各项工作的落实之年、深化之年、突破之年，我们要统筹兼顾、突出重点，着力抓好六方面工作：

(一)坚定不移调结构，打造保定产业升级版。把保定产业体系纳入京津冀协同发展版图，按照“5+2+5”路线图强势推进，积极构建与京津产业协调、优势互补，绿色、循环、低碳、高端的现代产业体系，增强发展硬实力。

推进服务业加快发展。坚持生产性服务业和生活性服务业[30]齐头并进，促进服务业扩容提质增效。培育壮大现代物流业。发展专业化、社会化的第三方物流，建设一批现代配送中心，抓好高碑店新发地、安国数字中药都[31]一期等京津产业转移物流项目。引进国内外大型物流企业，支持涞水与安博[32]、涿州与普洛斯[33]、清苑与中农批[34]等国内外物流名企合作，做大做强白沟现代物流产业聚集区。繁荣活跃现代金融业。优化金融生态环境，积极引进外埠金融机构，支持保定银行走出去，规范发展小额贷款公司、村镇银行等金融组织，加快构建多层次金融服务体系。抢抓国家支持资本市场发展新机遇，积极推进更多企业上市。创新投融资机制，探索政府与社会资本合作PPP模式[35]，建立城投基金和创投基金，扩大有效投资规模。建立健全地方金融监管体制，防范化解金融风险，严厉打击非法集资等金融违法犯罪行为。做大做强文化旅游业。着眼构建京津冀大旅游格局，发展休闲度假、生态观光、文化体验、医疗养生、体育健身等旅游业态，打造旅游精品项目。启动环首都“一核三区一带”[36]旅游规划，加快白洋淀休闲旅游度假、大白石山升级改造、易水湖综合开发[37]等旅游重点项目建设，支持白石山、清西陵、狼牙山、满城汉墓、天生桥、和道国际箱包城[38]等争创5A级景区。推进环京津体育健身休闲圈建设，打造全民健身户外活动基地。结合纪念抗战胜利70周年，举办“中国保定·世界反法西斯走廊之旅”[39]大型活动。加强旅游宣传推介，整治规范景区秩序，叫响保定旅游品牌。积极发展新兴服务业。推进“电子商务发展三年推进计划”[40]，引进知名电商，建设一批有影响力的电子商务平台。加快白沟、蠡县、满城、高阳、高新区等电商产业园(基地)建设，支持安国建立中药材电子商务期货交易平台，启动“淘宝特色·中国保定馆”[41]项目建设。加快发展科技咨询、服务外包、信息服务、文化创意等产业，打造朝阳北大街科技服务业隆起带[42]。规范发展快递服务业，推进快递服务向农村延伸。加强服务业统计监测，引导促进服务业健康发展。

做强工业经济支撑。坚持工业强市战略，巩固放大以现代装备制造业为主的产业特色，完善产业链条，推进“四化”融合发展，加快构建现代工业体系。发展壮大优势产业。汽车产业，突出抓好南部保定汽车科技城[43]、大王店汽车产业园和中兴、立中等重点项目，支持长城、风帆加快研发新能源汽车及动力电池，促进汽车及零部件产业集聚发展，持续打造“保定·中国汽车城”。新能源产业，突出抓好光伏规模化应用和风电技术研发，打造“太行光谷”，建设太行山百里绿色能源产业带。支持英利、国电联合动力等企业巩固行业领军地位，做大智能电网装备制造业。建材产业，重点发展节能门窗、环保涂料等产业，突出抓好高碑店国家建筑节能技术国际创新园[44]建设，办好第三届中国(高碑店)国际门窗博览会，加快推进徐水、博野晨阳水漆产业园[45]等项目建设。改造升级传统产业。认真落实省“十百千”工程[46]和千项技改项目，推动传统产业集群化、信息化、品牌化发展。纺织服装产业，重点推进高阳恒天天鹅绿色纤维产业园[47]、蠡县裘皮新城[48]等项目建设，发展新型纤维、高档面料、品牌服装等高附加值产品。食品产业，重点加快满城新希望天香乳制品基地[49]、定兴西麦扩能[50]、蠡县加多宝凉茶[51]等项目建设，发展农产品精深加工业，建设京津冀绿色食品加工基地。同时，推进县域特色产业二次创业，促进曲阳石雕、容城服装、雄县革塑、高蠡纺织、博蠡胶带、顺平肠衣、满城纸业、易县建材等特色产业上档升级，不断提升县域经济实力。加快培育新兴产业。生物医药产业，突出打造中药制药、生物医药生产基地，重点推进安国天士力、涞水悦康[52]、满城九孚生化[53]、北大未名集团[54]唐县古北岳生物医药等项目建设。电子信息产业，突出抓好信息消费试点城市建设，加快宽带网络、4G网络建设和推广应用，打造“智慧城市”。大力发展智能装备制造、航空航天和新材料等产业，力促满城乐凯新材料产业园[55]、涞水未来电子科技城[56]等项目加快建设，培育新的经济增长点。

推进工业发展，关键是让企业行健致远。我们将采取更加有力措施，继续对进入省“三个一百”[57]企业给予重点扶持，保障煤电油气运等要素供应，打造保定工业的旗舰企业。深入实施“双百小微企业晋级工程”[58]，落实“个转企、小升规” [59]扶持政策，促进中小微企业加快发展，力争新增规上企业200家以上。企业家是社会的宝贵财富，我们一定倍加关心爱护企业家，从今年开始定期举办“企业家大讲堂”，让他们开阔视野、提升素质，大胆创业、安心发展，在事业上得到更多支持，在社会上受到更多尊重!

提升农业现代化水平。遵循现代农业的发展方向，创新机制、创新措施，推进农业大市向农业强市转变。稳生产。严守耕地保护红线，继续实施高产创建行动，确保粮食播种面积稳定在1200万亩、总产500万吨以上。做强蔬菜水果、中药材、麻山药、苗木花卉等特色产业，扩大无公害农产品、绿色食品基地面积，建设一批奶牛、生猪、蛋鸡等规模化生产基地。瞄准京津中高端市场，完善农产品质量监测追溯体系，建设区域性农产品配送中心，培育一批全国知名农业品牌。抓园区。参照工业园区的模式抓好全市44个现代农业示范园区，促进园区提档升级。引进和培育一批农副产品加工企业，壮大一批农业产业化龙头。推进中国(涿州)农业科技创新园区[60]、京保田园谷[61]等重大项目建设，大力发展设施农业、生态农业和休闲观光农业。促增收。落实强农惠农政策，培育发展专业大户、家庭农场、农民股份合作社。组织开展各类培训，培育一大批懂技术、善管理、会经营的新型农民，稳定增加农民收入。强基础。抓好农村瓶颈道路改造和村内主街道硬化，新改建农村公路1000公里，健全城乡一体化客运网络。加快农田水利设施建设和农网升级改造，不断改善农业生产条件。解决78万人饮水不安全问题。新建33个乡镇邮政所，实现乡乡设所。推进农村面貌改造提升，加快白洋淀连片美丽乡村建设，努力改善农村人居环境，打造具有现代气息的幸福家园。

(二)坚定不移抓对接，增强跨越发展支撑力。以主动、务实、争先的精神抓机遇，把京津冀协同发展的事情做深、做细，把跨越发展的基础夯实、筑牢。

加强规划和交通对接。着眼落实国家京津冀协同发展规划，结合编制“十三五”规划，修订我市总体规划和重大专项规划，把京津冀协同发展的顶层设计本地化、具体化，为未来发展明方向、为率先融入争主动。推动交通互联互通，开工建设曲港、石津、西阜高速[62]保定段，推进荣乌高速保定段(大王店—涞源)建设；加快京白路(北京—白沟)等干线公路拓宽改造；推进廊涿城际(廊坊—涿州)及白沟支线铁路(白沟—首都二机场)前期工作，津保城际铁路(天津—保定)建成通车，构筑京津保快捷交通体系。

加快园区上档升级。统筹推进涿州新兴产业示范区、涞水首都功能疏解区[63]、首都新机场临空产业园等重点园区建设，实施城区老工业企业“退城入园”工程[64]，促进老企业焕发新生机。按照高端发展、错位发展的原则，明确34个园区功能定位，全面加快“五个一”建设，着力打造重大项目聚集地、京津产业转移承接地。实施品牌园区战略，重点建设一批产值超500亿元、超千亿元园区。

强化项目带动。安排省市重点项目300项左右，总投资6000亿元以上。继续落实重点项目领导分包、互看联查等推进机制，促进项目落地，提升建设质量。突出抓好与北京丰台区、朝阳区、中关村、亦庄经济开发区、天津滨海高新区等合作共建项目，确保年内取得突破。盘活土地资源，用足用好城乡建设用地增减挂钩、工矿废弃地复垦利用政策，做好耕地占补平衡，严明投资强度、容积率、回报率等标准，集约节约用地，让有限资源支撑更多项目建设。

深化对外开放。强化与京津科研院所、医院学校、政府机构的对接，重点在高新技术、新兴产业等方面，吸引更多企业、项目、人才落户。瞄准国际国内500强和行业领军企业，广泛开展上门招商、产业招商、园区招商、以商招商。年内组织到国内外重点地区举办主题招商活动，推进与中东欧、北美、大洋洲等地区交流合作，吸引世界目光关注保定。加快引进来、走出去步伐，鼓励支持企业国外建厂，扩大海外市场。

(三)坚定不移抓统筹，加快新型城镇化进程。围绕京津冀城市群重构，借势推动“大保定”建设，拓展空间，做大规模，提升品位，提高新型城镇化水平。

拉大城市框架。启动主城区行政区划调整，加强规划引领，做大城市发展空间。加快推进东部高铁片区、东湖文化中心、西湖文体中心、南部保定汽车科技城、北部低碳新城等板块建设，加速中心城区“东拓、西扩、南联、北延”，构建大城市格局。完成白洋淀大道、朝阳大街南延、北三环东延后续工程，加快实施复兴路西延、东风路西延、乐凯大街南延，规划启动阳光大街北延、七一路西延、北二环东延、长城大街南延和南二环、隆兴路改造等道路建设，打通断头路、卡脖路，加快小街巷改造，完善城市路网框架，改善市区交通环境。

提升城市品位。启动国家生态城市创建，加快市区主干道和城市出入口道路绿化提升，实施内外环水系提升工程，注重城市雕塑建设，做靓城市景观，让城市水绿交融、充满灵性。加快火车站和高铁站市政配套工程、标准化菜市场、公共自行车服务等功能设施建设，加强城市水、电、气等基础设施建设。加快三大片区拆迁改造，清真寺片区12月底前全部回迁，府河、西大园片区回迁安置房年底主体全部竣工。启动大世界、菊胡同等老旧小区改造，加快城中村连片开发。严厉打击违法违规建设，促进房地产市场平稳健康发展。持续推进城市管理6S行动，进一步巩固提升、深化延伸，推行数字化、网格化管理，全面改善城市环境，做到城净街美、规范有序，让城市更加宜居、更加文明、更加和谐。

加快城镇发展。加强城乡统筹规划，抓好新型城镇化试点，打造中小城市群。坚持产业、文化、生态融合发展，推进“多规合一”[65]，鼓励支持各县改造提升老城区、开发建设新城区，促进县城向各具特色、功能完善的小城市发展。推进“七个一”工程，加快博物馆和河道景观建设，做好园林景观设计，塑造县城特色风貌。加快县(市)南水北调地表水厂和输水管道建设，确保明年汛期后用上长江水。望都、顺平、满城、高阳等11个县创建省级园林县城，力争年内全覆盖。建设一批工业强镇、旅游名镇、特色小镇，完善基础设施，增强服务功能。落实户籍改革制度，加快农业人口转移，让更多农民过上城里人的日子。

(四)坚定不移治污染，力促生态环境明显改善。保护良好的生态环境、增强保定的生态魅力，是维护群众生命健康的应尽之责，也是京津冀协同发展至关重要、必须做好的事情。我们要以背水一战的决心，用更有效的办法，以更大的气力，建设天蓝、水净、地绿的美好家园。

守住蓝天。实施大气污染深入治理三年(2015-2017年)行动，以改善空气质量为目标，以优化能源结构为重点，抓住燃煤和扬尘两大关键，坚决打赢治霾攻坚战。一是集中供热。加强市场化运作，在市区、县城和重点园区，加快建设热电联产、集中供热项目和配套管网。年内淘汰所有10吨以下工业燃煤锅炉。二是能源优化。市区城中村全部实现洁净型煤配送，或改用生物质、电、地热等清洁能源。推广“雄县模式”[66]，科学开发利用地热资源。加强散煤专项治理，市区新建15家洁净型煤生产配送中心，全市禁烧劣质煤。严禁焚烧秸秆，大力推进秸秆综合利用。三是抑制扬尘。继续开展好洗城净天行动，突出抓好城乡结合部的环境整治和城市外环路的道路扬尘管控，完成砂石开采、小灰窑行业的停产整治、转型升级。实施“绿色建筑”节能工程。全部淘汰黄标车。引进环境污染第三方治理[67]，探索用市场化手段治污，推进环境污染治理专业化、规模化、集约化。多措并举下狠劲，较真叫板出重拳，力争在2-3年内使空气质量明显改善，甩掉重污染城市的“黑帽子”，还人民一片蓝天。

保护碧水。实行最严格的水资源管理制度，加大水生态保护和污染防治力度，完成市区污水处理厂一级A改造，完善徐水、高碑店、容城等县(市)城镇污水管网，提高污水处理率。推进白洋淀生态环境综合治理，打造河北省首个国家湿地公园。加强饮用水水源地生态保护，坚决遏制地下水超采，合理用好地上水，推进引黄入冀补淀工程，努力提升水资源承载能力。

植树增绿。落实《山水林田湖生态修复规划》，实施一批重大生态修复工程。抓好京津保生态过渡带建设，推进造林绿化三年攻坚行动，以“三网四带”[68]建设为重点，完成人工造林和封山育林70万亩，森林覆盖率达到26.7%。严格保护林地和森林资源。抓好矿山整治，推进矿山园区化、生产规范化，加快京昆等高速沿线山体修复，年内完成97个矿山综合治理。

铁腕监管。加强工业污染治理和环境监管。建立市县乡村四级网格化监管系统，形成群防群治工作格局。落实部门监管责任和企业主体责任，健全举报制度，加强社会监督，严格依法惩处，重拳打击违法排污，严厉查处破坏环境案件。

(五)坚定不移抓改革，营造全民创新创业氛围。改革是一场革命，创新是一种提升，全民创业才能焕发城市激情。我们要以改革生动力、以创新增活力、以创业强实力，为融入京津、加快发展提供强大引擎。

全面深化改革。加快行政体制改革。进一步简政放权，最大限度为各类市场主体松绑，再取消和下放一批行政审批事项，全面清理非行政许可审批和前置审批，完善事中事后监管，推进网上一体化审批和联合审批。推行县以下机关公务员职务与职级并行制度。年内完成机关公务用车制度改革。加快经济体制改革。加强经营性国有资产统一监管，把监管责任落实到位、企业自主经营权保障到位。完善国有企业法人治理结构，探索建立国有企业信息披露制度，严肃国有产权交易制度。发展混合所有制经济，优化国有资本股权结构。分步实施市属商贸流通企业破产改制。整合盘活事业单位存量国有资产。推进农村产权制度改革，稳步开展土地承包经营权确权登记，引导和规范土地经营权有序流转，发展多种形式的适度规模经营，培育壮大各类新型农业经营主体。落实工商企业“三证合一”登记制度[69]，扩大“营改增”试点范围。加快供销社综合改革。加快管理体制改革。实行全面规范、公开透明的预算管理制度，深化绩效预算管理改革。推进水务一体化改革。加快开发区管理体制改革，赋予高新区更多体制机制创新自主权，在白沟新城及雄县、容城等县(市)探索区域统筹发展新机制。同时，继续抓好医药卫生体制改革，深化水、电、气等资源性产品价格改革。

增强创新能力。把今年作为“创新能力提升年”，加大“科技进步县”考核力度，建设国家创新型试点城市。搭建平台。举全市之力推进白洋淀科技城建设，突出生态与科技主题，完善相关规划，创新体制机制，加快启动核心区特别是起步区建设，争创国家自主创新示范区，打造京津冀创新成果转化基地、世界级的科技创新产业平台。进一步抓好中关村·保定创新中心、涞源白石山中美科技国际创新园、高碑店国家建筑节能技术国际创新园等重点创新型园区建设，完成9个示范县(区)科技企业孵化器[70]建设。推进英利科技工业园区[71]建设，打造京津冀区域最具影响力的新型城镇化样#from 本文来自学优网http://www.gkstk.com end#板。激活主体。以长城、英利、晨阳等骨干企业为重点，加大自主创新力度，力争年内新认定3家省级以上企业技术中心、工程实验室，新建2家省级以上工程技术研究中心、重点实验室。实施高新技术企业培育工程，年内新增15家以上。组建联盟。进入新的一年，我市成立了光电网、新能源应急产业和第三代半导体三个技术创新战略联盟[72]。围绕光伏发电、智能电网、新能源汽车、通讯制造与服务、中药现代化等重点优势领域，将再组建一批产业技术创新联盟。发挥驻保高校作用，进一步加强产学研合作，引导创新要素聚集，加快“保定制造”向“保定智造”、“保定创造”转变。广纳人才。实施人才强市战略，完善引进高层次人才的政策规定和奖励办法，在全社会营造尊重知识、尊重技术、尊重人才的浓厚氛围。

鼓励全民创业。围绕大众创业、万众创新，再推出一批创业扶持政策，努力培养造就一批本土创业大军，让创业者奋斗有回报、发展有希望。加快科技成果产权制度改革，推行股权和分红激励等政策，支持科技人员创办领办科技型企业。加强创业培训，整合利用大中专院校和社会培训资源，建立创业培训基地，为创业者提供全方位服务，让众多“创客”[73]自由创业、能够成业，使千千万万小微企业成长壮大、活力迸发。

(六)坚定不移惠民生，让百姓共享发展成果。坚持公共财政向民生领域倾斜，鼓励社会资本发展社会事业。更加注重保障基本民生，更加关注低收入群众生活，更加维护社会公平正义，逐步缩小与京津的公共服务差距。

加强社会保障。积极稳定扩大就业，统筹解决好高校毕业生、农村转移劳动力、退役军人等重点人群就业问题，确保城镇新增就业8.3万人。建立更加公平可持续的社会保障制度，推进社保扩面，提高企业退休人员和城乡居民养老金、工伤保险待遇标准，推动医疗保险异地就医即时结算。城乡居民基本医疗保险参保率达到95%以上，新农合、城镇居民医保人均政府补助提高到380元。城乡居民低保标准保持与全国平均水平同步增长。推进机关事业单位养老保险制度改革。实施临时救助制度，及时帮扶困难群众。健全养老服务体系，新建一批社区老年人日间照料中心和农村互助幸福院，推进社会化养老，让更多人老有所依、老有所养。

发展社会事业。坚持教育优先发展，推进义务教育均衡发展，支持民办教育加快发展。强力推进三个“教育提升三年行动计划”，力争年内开工建设5所市区中小学、10所山区寄宿制学校，新改扩建43所县城中小学。大力发展职业教育，加快迁建保定职业技术学院。提高医疗卫生水平，做好重大疾病防控工作，完成市儿童医院迁建，加快市妇幼保健院和市第一中心医院改扩建，抓好县级中医院和妇幼保健院(站)建设。积极推进与凤凰医疗集团合作，鼓励社会资本参与公立医院改革，健全综合医疗服务体系。坚持计划生育基本国策，落实各项计生惠民政策。加强国防教育和后备力量建设，推动军民融合发展。做好妇女儿童、残疾人、气象防震减灾、民族宗教、外事侨务、史志档案等工作。

弘扬保定文化。推进文化名城建设，保护修缮和综合利用直隶总督署、淮军公所、清河道署，加快西大街业态整治。加强文物古迹、古村落和非物质文化遗产保护与传承。实施文艺精品工程，创作一批经典剧目。推进与中国东方演艺集团等国家级院团合作，积极开展“深入生活、扎根人民”和“文化润古城”等主题实践活动。广泛开展全民健身运动，继续办好空竹艺术节等大型品牌文体活动，推动学校、企业等体育设施向社会开放。

推进扶贫攻坚。推行精准扶贫，发展富民产业。推进春雨行动[74]、雨露计划[75]，广泛开展社会扶贫。对新建档立卡的贫困村和贫困户，逐村逐户制定脱贫发展规划，重点分包、定点帮扶、动态管理，切实提高扶贫效果。加强整村推进，推广涞水整乡脱贫和阜平葫芦峪等市场化扶贫模式，大力发展阜平电商、曲阳光伏等扶贫新模式，支持发展家庭特色手工业，让贫困群众尽快增收脱贫。年内确保200个村、15万人脱贫，涞水、望都、博野率先达到脱贫标准。

加强社会治理。落实安全生产“党政同责、一岗双责”，加大重点行业、重点领域安全生产整治力度，坚决遏制重特大事故发生。建立完善覆盖全市的县、乡、村食品药品安全监管体系，夯实基层基础，强化技术支撑，深入开展食品、药品、保健品和生鲜农产品市场专项整治，严厉打击食品药品违法犯罪，确保人民群众饮食用药安全。畅通信访渠道，推行阳光信访，抓好访诉分离，引导依法信访，用法治思维化解社会矛盾。加强公共安全管理，健全安全防控体系。按照“一年四打”部署，依法打击各类违法犯罪，为百姓创造和谐稳定、安全有序的生产生活环境。

各位代表!群众利益无小事，民生问题大于天。今年，我们广泛征求社会意见，本着民生优先、积极而为、量力而行、办就办好的原则，拟定了10个方面65项惠民事项。今后工作中，我们一定坚持以改善民生为根本，始终把人民的冷暖放在心上，全心全意增进人民福祉，广泛汇集跨越发展正能量，用扎扎实实的工作和实实在在的业绩，回应人民群众对美好生活的新期待!

三、建设为民务实清廉的法治政府

面对全面建成小康社会、全面深化改革、全面推进依法治国的新形势，面对艰巨繁重的新任务，我们必须不断加强政府自身建设，不断提高政府公信力和执行力，不断提高政府工作法治化水平。

坚持科学理政。更加注重学习、锤炼本领，把握发展大势，保持战略定力，增强在新常态下推进工作的能力和水平。广泛听取社会各界的意见建议，安排部署工作讲科学、不蛮干，使之更加符合客观实际、更加体现群众意愿。注重发现苗头性、倾向性问题，未雨绸缪，提前应对，牢牢把握加快发展的主动权。

坚持依法行政。法定职责必须为，法无授权不可为。坚持权责统一，加强权力运行的监督制约，完善“四份清单、一个标准”，强化执行和督查。规范执法行为，健全行政处罚自由裁量权基准制度。严格依法按程序决策，建立健全合法性审查机制，建立重大决策终身责任追究制度及责任倒查机制。坚持为政职业操守，做到言必信、行必果，以政府诚信带动社会诚信。支持配合人大行使立法权，主动接受人大法律监督、政协民主监督和社会公众监督，推进政务“五公开”[76]，让政府权力在阳光下运行。

坚持高效施政。干字当头、掷地有声，定下的事情，抓紧实施，及早完成；部署的工作，一抓到底，确保实效。坚持以目标倒逼进度、以督查强化落实，使各项工作高速运转、高效运行。大力惩治庸、懒、散、贪、拖等不良作风，扎实开展好“深化整改、从严治理、提高效能、促进发展”专项行动，严厉查处破坏发展环境、损害群众利益的行为。进一步完善“三级平台[77]、两个代办”制度，强化市县两级政务服务中心硬件建设，拓展服务功能，决不让老百姓办事摸不着门、跑累了腿。

坚持清廉从政。巩固深化教育实践活动成果，坚持不懈反“四风”,严格执行中央八项规定和廉洁自律各项规定，认真落实“三严三实”[78]要求，推进作风建设常态化。坚持勤俭节约办事，严控“三公”经费和一般性支出。加强反腐倡廉，推进源头治理、制度反腐，坚持“一案双查”[79]，严厉查处违纪违法案件，全力营造风清气正的政务环境。我们要干干净净地干事，不能碌碌无为的干净，对违法违纪的贪污腐败分子坚决查处，对为官不为的政府公职人员决不姑息!

各位代表!梦想承载希望，奋斗开创未来。今天的保定，站在了新的历史起点，面临着跨越发展的重大机遇，天时、地利、人和俱备，机不可失、时不我待。让我们在省委、省政府和市委的坚强领导下，鞠躬尽瘁为百姓，殚精竭虑谋发展，与1000多万勤劳智慧的保定人民携手共进，解放思想、勇于担当，拼搏进取、奋发作为，以实际行动回答好“保定四问”[80]，全力开创京津冀协同发展新局面，为建设京畿强市、善美保定，打造环京津新的发展增长极而努力奋斗!
